# Association of the Reduced Levels of Monocyte Chemoattractant Protein-1 with Herpes Zoster in Rheumatoid Arthritis Patients Treated with Janus Kinase Inhibitors in a Single-Center Cohort

**DOI:** 10.3390/microorganisms12050974

**Published:** 2024-05-12

**Authors:** Po-Ku Chen, Yi-Ming Chen, Hsin-Hua Chen, Tsai-Ling Liao, Shih-Hsin Chang, Kai-Jieh Yeo, Po-Hao Huang, Der-Yuan Chen

**Affiliations:** 1 Rheumatology and Immunology Center, China Medical University Hospital, Taichung 404, Taiwan; pago99999@gmail.com (P.-K.C.); sherry61976@gmail.com (S.-H.C.); dryeokj@gmail.com (K.-J.Y.); u402082@gmail.com (P.-H.H.); 2 College of Medicine, China Medical University, Taichung 404, Taiwan; 3 Translational Medicine Laboratory, Rheumatology and Immunology Center, China Medical University Hospital, Taichung 404, Taiwan; 4 Division of Translational Medicine, Department of Medical Research, Taichung Veterans General Hospital, Taichung 407, Taiwan; blacklark@gmail.com; 5 School of Medicine, National Yang Ming Chiao Tung University, Taipei 112, Taiwan; 6 Department of Post-Baccalaureate Medicine, College of Medicine, National Chung Hsing University, Taichung 402, Taiwan; shc5555@vghtc.gov.tw (H.-H.C.); tlliao1972@gmail.com (T.-L.L.); 7 Department of Industrial Engineering and Enterprise Information, Tunghai University, Taichung 407, Taiwan; 8 Big Data Center, National Chung Hsing University, Taichung 402, Taiwan; 9 Department of Medical Research, Taichung Veterans General Hospital, Taichung 407, Taiwan; 10 Ph.D. Program in Translational Medicine, Rong Hsing Research Center for Translational Medicine, National Chung Hsing University, Taichung 402, Taiwan; 11 Institute of Clinical Medicine, Chung Shan Medical University Hospital, Taichung 402, Taiwan

**Keywords:** anti-interferon-γ autoantibodies, herpes zoster, monocyte chemoattractant protein-1, IFN-γ inducible protein-10, Janus kinase inhibitors, rheumatoid arthritis

## Abstract

Anti-interferon (IFN)-γ autoantibodies are linked to varicella zoster virus (VZV) infection. Given the elevated risks of herpes zoster (HZ) in rheumatoid arthritis (RA) patients treated with Janus kinase inhibitors (JAKis), we aimed to examine the relationship between anti-IFN-γ autoantibodies with HZ development in JAKi-treated patients. Serum titers of anti-IFN-γ autoantibodies, plasma levels of IFN-γ, monocyte chemoattractant protein-1 (MCP-1), and IFN-γ-inducible protein-10 (IP-10) were measured by ELISA. Among the 66 enrolled RA patients, 24 developed new-onset HZ. Significantly lower MCP-1 levels were observed in patients with HZ compared to those without (median, 98.21 pg/mL, interquartile range (IQR) 77.63–150.30 pg/mL versus 142.3 pg/mL, IQR 106.7–175.6 pg/mL, *p* < 0.05). There was no significant difference in anti-IFN-γ titers, IFN-γ levels, or IP-10 levels between patients with and without HZ. Three of 24 patients with HZ had severe HZ with multi-dermatomal involvement. Anti-IFN-γ titers were significantly higher in patients with severe HZ than in those with non-severe HZ (median 24.8 ng/mL, IQR 21.0–38.2 ng/mL versus 10.5 ng/mL, IQR 9.9–15.0 ng/mL, *p* < 0.005). Our results suggest an association between reduced MCP-1 levels and HZ development in JAKi-treated RA patients. High-titer anti-IFN-γ autoantibodies may be related to severe HZ in these patients.

## 1. Introduction

Interferon (IFN)-γ, a type II IFN (IFN-II), is essential for the host’s defense against infection with intracellular pathogens [1,2]. After STAT1 phosphorylation, IFN-γ can activate the transcription of genes of cytokines or chemokines that play a crucial role in antimicrobial activity. Monocyte chemoattractant protein-1 (MCP-1), a C-C-type chemokine induced by IFN-γ in lymphocytes, promotes the migration of monocytes to sites of inflammation in response to infectious pathogens such as COVID-19 and tuberculosis [3,4]. Gaudreault et al. revealed that the Epstein–Barr virus infection of human monocytes could induce the release of MCP-1 [5]. Moreover, IFN-γ-induced chemokines such as IFN-γ-inducible protein-10 (IP-10, also known as CXCL10) may amplify inflammatory responses. Choi et al. demonstrated that varicella zoster virus (VZV) infection could increase the production of interleukin (IL)-6, IL-8, and IP-10 [6]. Although the exact mechanisms behind the autoantibody formation against IFN-γ (anti-IFN-γ autoAbs) remain unclear, several studies have shown that these autoAbs have a suppressive effect on IFN-γ signal transduction [7,8]. Therefore, neutralizing anti-IFN-γ autoAbs is associated with increased risks of opportunistic infections (OIs), including VZV infection [7,8,9,10]. Hong et al. revealed that anti-IFN-γ autoAb titers were closely related to the severity of infections, reflecting the biological activity of anti-IFN-γ autoAbs [11]. Anti-IFN-γ autoAbs have also been observed in autoimmune inflammatory diseases such as systemic lupus erythematosus (SLE) and rheumatoid arthritis (RA) [12,13], despite the unclear clinical impact.

RA, an inflammatory disease, is characterized by persistent synovitis, bone destruction, and poor life quality [14]. Epidemiological studies reveal that RA patients have an elevated risk of HZ compared to the general population [15,16,17]. Targeting the complex pathogenesis of RA, emerging new agents are available for the treatment of this disease, including biologic disease-modifying anti-rheumatic drugs (bDMARDs) and targeted synthetic DMARDs (tsDMARDs) such as Janus kinase inhibitors (JAKi) [18]. Although their therapeutic efficacies are comparable to bDMARDs, JAKis are associated with a significantly increased incidence of HZ in clinical trials and long-term extension studies [19,20]. The involvement of the JAK/signal transducer and activator of transcription (STAT1) pathway in the anti-viral actions of IFN-γ [21,22] may explain an increased HZ risk associated with JAKi therapy. However, the associations between anti-IFN-γ autoAbs and the risk of new-onset HZ has yet to be explored in RA patients treated with JAKis.

This retrospective study aimed to compare the difference in serum titers of anti-IFN-γ IgG between RA patients with and without new-onset HZ during the period of 4.1 ± 1.8 years after initiation of JAKi therapy. We also determined the plasma levels of IFN-γ, MCP-1, and IP-10, which were related to the IFN-γ signaling-mediated STAT1 transactivation. Finally, the associations of anti-IFN-γ IgG with RA activity parameters and proinflammatory cytokines/chemokines were examined in JAKi-treated patients.

## 2. Materials and Methods

### 2.1. Patients and Study Design

In this retrospective and single-center study, we enrolled patients who had RA according to the American College of Rheumatology/European League Against Rheumatism criteria [23] with or without the development of new-onset HZ after starting JAKi therapy. The eligible criteria for inclusion were as follows: (1) age at study entry older than 20 years, (2) persistence of active disease despite treatment with conventional synthetic DMARDs (csDMARDs) or bDMARDs for at least 6 months, (3) a follow-up period of 4.1 ± 1.8 years after starting therapy with a standard dose of JAKi, and (4) ethnically unrelated Han Chinese. The exclusion criteria were mainly current infection, malignancy, or severe hepatic impairment before starting JAKi therapy in this study. Patients who received a zoster vaccination after starting JAKi therapy were also excluded. The JAKis used included tofacitinib (11 mg once daily, *n*= 50), baricitinib (4 mg once daily, *n* = 4), and upadacitinib (15 mg once daily, *n* = 12), either as monotherapy or in combination with methotrexate (MTX), leflunomide, or hydroxychloroquine. RA disease activity was assessed using the 28-joint disease activity score–erythrocyte sedimentation rate (DAS28-ESR) [24], with active disease defined as a DAS28 score of 3.2 or higher. Blood samples were obtained before the emergence of new-onset HZ in RA patients receiving JAKi therapy. This study received approval from the Institutional Review Board of Chinese Medicine University Hospital (CMUH110-REC1-086), and informed consent was obtained from each participant in accordance with the Declaration of Helsinki.

### 2.2. Diagnosis and Severity of HZ

HZ was diagnosed based on rheumatologists’ clinical assessment and the use of anti-viral therapy, according to the medical records. This methodology has been validated in previous large studies [25], demonstrating a positive predictive value of 97.5% in identifying HZ cases [25]. The history of HZ before starting JAKi treatment was extracted from patients’ medical records and was confirmed by phone calls. Disseminated HZ (a diffuse rash spanning more than six dermatomes), HZ with multi-dermatomal involvement (2 non-adjacent or 3–6 adjacent dermatomes), central nervous system involvement such as encephalitis, or the involvement of non-skin organs were considered to be severe HZ.

### 2.3. Clinical Phenotypes

The clinical data collected from RA patients included demographic data, disease duration, disease activity, positivity for rheumatoid factor (RF) or anti-citrullinated peptide antibodies (ACPA), history of HZ prior to initiating JAKi therapy, the concomitant use of corticosteroids or csDMARDs, and smoking status.

### 2.4. Determination of RF and ACPA

Serum levels of RF-immunoglobulin (Ig)M were determined using the IMMAGE^®^ Immunochemistry Systems and Calibrator 5 Plus (Beckman Coulter Ireland Inc., Mervue Business Park, Mervue, Galway, Ireland). Levels <20 IU/mL were considered negative. The ACPA-IgG levels were determined using the EliA™ technique (POhadia 250; Thermo Fisher Scientific, Uppsala, Sweden). Levels were considered negative if <7 U/mL, equivocal if 7–10 U/mL, and positive if >10 U/mL.

### 2.5. Determination of Serum Titers of Anti-IFN-γ IgG with ELISA

Each whole-blood sample (ten milliliters) was collected in a tube containing EDTA (BD Biosciences, San Jose, CA, USA) and then centrifuged at 2000 rpm for 10 min. Serum titers of IgG autoantibodies to IFN-γ were determined with ELISA (cat. abx585481, Abbexa Ltd., Cambridge, UK), according to the manufacturer’s instructions.

### 2.6. Determination of Plasma Levels of IFN-γ, MCP-1, and IP-10

We used commercial ELISA kits to determine the plasma levels of MCP-1 (DY279, R&D Systems, Minneapolis, MN, USA), IFN-γ (DY285B, R&D Systems, Minneapolis, MN, USA), and IP-10 (DY266, R&D Systems, Minneapolis, MN, USA) according to the manufacturer’s instructions.

### 2.7. Statistical Analysis

The results were presented as numbers (percent), mean ± standard deviation (SD), or median (interquartile range [IQR]). The *p*-values were calculated using the chi-squared test for between-group differences in categorical variables. The Mann–Whitney U and Fisher exact t-test were used for between-group comparisons of numerical variables. The correlation coefficient was obtained through the nonparametric Spearman’s rank correlation test. The missing values were excluded from the statistical analysis. A two-sided probability of less than 0.05 was considered significant.

## 3. Results

### 3.1. Comparison of Clinical Characteristics, Laboratory Data, and HZ-Related Status between RA Patients with and without New-Onset HZ

Among these 66 RA patients who received JAKi therapy during the follow-up period of 4.1 ± 1.8 years, 24 had new-onset HZ as the case group. Forty-two RA patients without the development of new-onset HZ during the JAKi therapeutic period were used as the control group. Of the 24 patients with HZ after starting JAKi therapy, severe HZ was observed in 3 patients who had multi-dermatomal involvement. RA patients with new-onset HZ had significantly longer disease duration, higher doses of concomitant corticosteroids, and a higher proportion of high-dose corticosteroids compared to those without HZ after starting JAKi therapy. There were no significant differences in the age at study entry, the proportion of females, the proportion of prior HZ history, baseline disease activity, the positive rate of RF or ACPA, the proportion of JAKi monotherapy, the concomitant csDMARDs, different JAKis, or smoking status between RA patients with and without new-onset HZ (Table 1).

### 3.2. Comparison of the Titers of Anti-IFN-γ IgG, Plasma Levels of IFN-γ, MCP-1, and IP-10 between RA Patients with and without HZ

**Figure 1:**
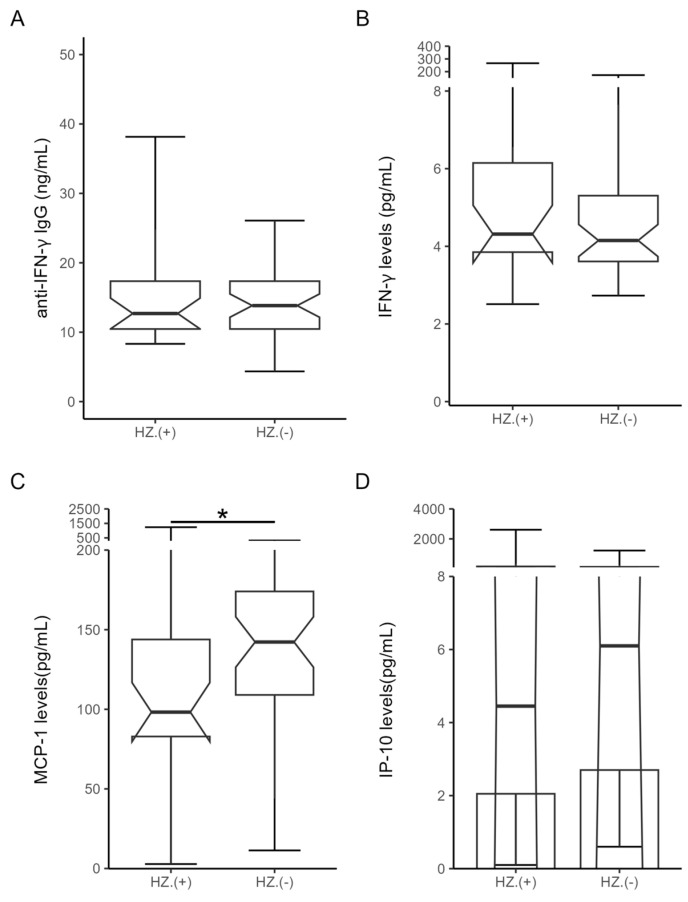
Comparison of (**A**) the titers of anti-IFN-γ IgG, (**B**) IFN-γ, (**C**) MCP-1, and (**D**) IP-10 between RA patients with and without HZ. The titers of anti-IFN-γ IgG and plasma levels of IFN-γ, MCP-1, and IP-10 were determined by enzyme-linked immunosorbent assay (ELISA). * Data are presented as the notched box and whisker plot. The horizontal line within the box indicates the median value for each group. * *p* < 0.05, determined by the Mann–Whitney U test. Anti-IFN-γ IgG, anti-interferon-γ IgG; IFN-γ, interferon-γ; MCP-1, monocyte chemoattractant protein-1; IP-10, IFN-γ inducible protein-10; RA: rheumatoid arthritis.; HZ, herpes zoster.

As illustrated Figure 1C, significantly lower levels of MCP-1 were observed in RA patients with new-onset HZ compared to those without HZ (median, 98.21 pg/mL, interquartile range (IQR) 77.63–150.30 pg/mL versus median 142.3 pg/mL, IQR 106.7–175.6 pg/mL, *p* < 0.05). There was a trend of lower IP-10 levels (Figure 1D) observed in RA patients with HZ compared to those without HZ (mean, 4.45 pg/mL versus 6.10 pg/mL, *p* = 0.48, Figure 1D). However, there was no significant difference in the titers of anti-IFN-γ IgG (median 12.69 ng/mL, IQR 10.46–17.35 ng/mL versus median 13.83 ng/mL, IQR 10.46–17.96 ng/mL, Figure 1A), or plasma levels of IFN-γ (median, 4.32 pg/mL, IQR 3.69–6.42 pg/mL versus 4.15 pg/mL, IQR 3.58–5.36 pg/mL, *p* = 0.56, Figure 1B) between RA patients with and without new-onset HZ.

### 3.3. Comparison of the Titers of Anti-IFN-γ IgG, Plasma Levels of IFN-γ, MCP-1, and IP-10 between RA Patients with Severe and Non-Severe HZ

**Figure 2:**
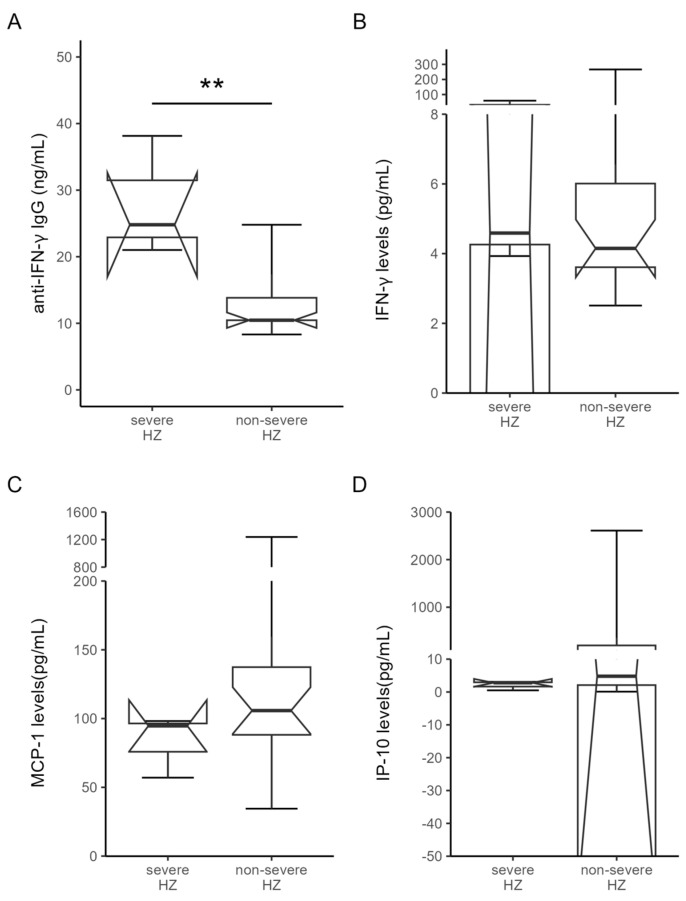
Comparison of (**A**) the titers of anti-IFN-γ IgG, (**B**) IFN-γ, (**C**) MCP-1, and (**D**) IP-10 between RA patients with severe HZ and non-severe HZ. Among 24 patients with new-onset HZ, three patients had severe HZ presenting with multi-dermatomal involvement. Data are presented as the notched box and whisker plot. The horizontal line within the box indicates the median value for each group. ** *p* < 0.005, determined by the Mann–Whitney U test. Anti-IFN-γ IgG, anti-interferon-γ IgG; IFN-γ, interferon-γ; MCP-1, monocyte chemoattractant protein-1; IP-10, IFN-γ inducible protein-10; RA: rheumatoid arthritis.; HZ, herpes zoster.

Among 24 patients with new-onset HZ after starting JAKi therapy, three patients had severe HZ presenting with multi-dermatomal involvement. As illustrated in Figure 2A, significantly higher titers of anti-IFN-γ IgG were observed (median 24.8 ng/mL, IQR 21.0–38.2 ng/mL versus median 10.5 ng/mL, IQR 9.9–15.0 ng/mL, *p* < 0.005). However, there was no significant difference in the plasma levels of IFN-γ (median, 4.59 pg/mL, IQR 3.93–59.03 pg/mL versus 4.15 pg/mL, IQR 3.61–6.29 pg/mL, *p* = 0.50, Figure 2B), MCP-1 (median, 94.68 pg/mL, IQR 57.03–98.21 pg/mL versus median 105.80 pg/mL, IQR 82.91–148.50 pg/mL, Figure 2C), or IP-10 (median 2.80 ng/mL, IQR 0.50–3.10 ng/mL versus median 4.80 ng/mL, IQR 2.00–204.60 ng/mL, Figure 2D) between RA patients with severe and non-severe HZ.

### 3.4. Association of Serum Titers of Anti-IFN-γ IgG with RA Disease Activity and Inflammatory Parameters

**Figure 3:**
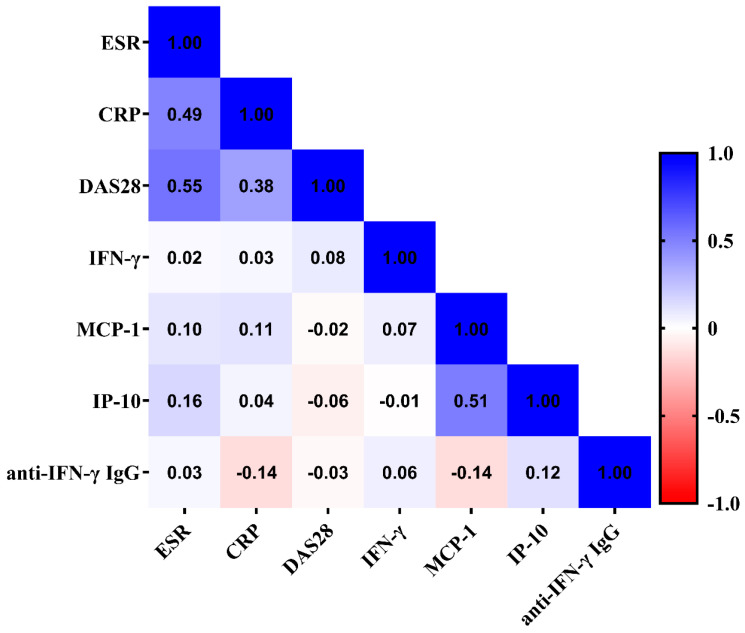
Correlation matrix between anti-IFN-γ IgG titers and RA activity parameters, cytokines, and chemokines. The correlation coefficient was obtained through the Spearman’s rank correlation test. The bar showed correlation coefficients ranging from −1 to +1”. Anti-IFN-γ IgG, anti-interferon-γ IgG; ESR, erythrocyte sedimentation rate; CRP, C-reactive protein; DAS28, the 28-joint disease activity score; IFN-γ, interferon-γ; MCP-1, monocyte chemoattractant protein-1; IP-10, IFN-γ inducible protein-10; RA: rheumatoid arthritis.

As shown in Figure 3, there was a trend of inverse correlation between the anti-IFN-γ IgG titers and plasma levels of MCP-1. However, there was no significant correlation between anti-IFN-γ IgG titers and RA disease activity, reflected by DAS28 scores, and inflammatory parameters in RA patients.

## 4. Discussion

Increasing evidence suggests a close link between anti-IFN-γ autoAbs or chemokines and OIs [7,8,9,10,11,26,27], yet their relationship in RA patients with new-onset HZ has not been explored. Compared to RA patients without HZ development, our JAKi-treated patients with new-onset HZ had a longer disease duration, higher doses of corticosteroids, and a higher proportion of high-dose corticosteroids used. We also reveal significantly lower levels of MCP-1, but not anti-IFN-γ IgG, in RA patients with VZV reactivation after starting JAKi therapy. It is worth mentioning that significantly higher titers of anti-IFN-γ IgG was observed in three patients with severe HZ presenting with multi-dermatomal involvement than in those with non-severe HZ. These findings suggest that low-level MCP-1 is associated with HZ development, and high-titer anti-IFN-γ IgG is probably related to severe HZ in RA patients after the initiation of JAKi therapy. Further validation in studies with a larger sample size is needed.

Although anti-cytokine autoAbs have been observed in immune-mediated inflammatory diseases [12,26,27], whether anti-IFN-γ autoAbs in these diseases contribute to the susceptibility to infections remains unclear. Recently, Chen et al. revealed significantly higher titers of anti-IFN-γ IgG in SLE patients with severe infections compared to those without infections [27]. They also identified anti-IFN-γ IgG as a significant predictor for developing severe infections in SLE patients [27]. Anti-IFN-γ IgG-positive SLE patients were also more susceptible to mycobacterial and fungal infections compared to those without anti-IFN-γ IgG [27]. Although we demonstrated no significant difference in the serum titers of anti-IFN-γ IgG between RA patients with and without new-onset HZ, significantly higher titers of anti-IFN-γ IgG were observed in RA patients with severe HZ than in those with non-severe HZ. These observations support the findings reported by Hong et al. that anti-IFN-γ IgG titers were strongly associated with the severity of infections [11].

In the biological role of IFN-γ signaling against infection with intracellular pathogens [1,2], IFN-γ could activate the transcription of genes of proinflammatory cytokines or chemokines after STAT1 phosphorylation. Previous studies demonstrated that MCP-1 could promote the chemotaxis of monocytes to sites of inflammation in response to infectious pathogens [3,4]. Gaudreault, E. observed that Epstein–Barr virus could increase MCP-1 secretion in monocytes [5]. Krisnawati et al. revealed that treatment with non-tuberculous mycobacterial infection (NTM) patients’ sera significantly blocked the IFN-γ-induced production of IFN-γ, MCP-1, and IP-10 [28]. Considering that the neutralizing effects of anti-IFN-γ autoAbs on the IFN-γ signaling pathway are mediated by targeting STAT1 transactivation and chemokine production, we also revealed significantly lower levels of plasma MCP-1 in RA patients with new-onset HZ compared to those without HZ. Given that MCP-1 could contribute to the anti-microbial inflammatory response by attracting monocytes and T lymphocytes [29], the low level of MCP-1 may increase infection with intracellular pathogens such as varicella zoster virus. According to the evidence from other studies [26,27,28,29,30] and ours, high-titer anti-IFN-γ IgG may reduce antimicrobial activity, at least partly, by counteracting the IFN-γ-mediated production of chemokines. Although anti-IFN-γ IgG could neutralize IFN-γ, the non-significant difference in plasma IFN-γ levels between RA patients with and without HZ was probably due to the small sample size in our study.

It has been proposed that the development of anti-cytokine antibodies may be part of an immune regulatory response to inflammation or due to abundant cytokine exposure [31,32]. Gupta et al. demonstrated an association of anti-IFN-γ autoAbs with SLE disease activity, rather than with opportunistic infections [12]. Chen et al. also revealed a positive correlation between anti-IFN-γ IgG titers and SLE disease activity [27]. However, we revealed no significant correlation between anti-IFN-γ IgG titers and disease activity or inflammatory parameters in the RA group. The discrepancy may be due to the differences in the diseases and patients’ characteristics, the methods for detecting anti-IFN-γ IgG, and the medications used.

Despite the novel findings, there are some limitations of this study. The retrospective nature of our study did not allow for obtaining all of the needed information from the enrolled patients. The lack of a significant difference in anti-IFN-γ IgG titers between RA patients with and without new-onset HZ might be due to the insufficient number of patients with new-onset HZ. In addition, the collection of blood from JAKi-treated patients cannot take place during the acute infection phase of HZ. Given the higher dose of corticosteroids prescribed in RA patients with HZ than in those without HZ (Table 1), the titers of anti-IFN-γ IgG might also be influenced by the therapeutic agents in the present study. The non-significant correlations between the anti-IFN-γ IgG titers and RA activity or the downstream cytokines/chemokines may be due to the small sample size of our RA cohort. Finally, HZ development may result from the inherited mutations of IFN-γ-signaling-related genes, which were not evaluated in our RA patients. Therefore, a large-scale study with sufficient statistical power is needed to validate this finding and support its clinical implication. Considering the limited number of patients with severe HZ, future long-term studies that enroll more JAKi-treated patients are also needed.

## 5. Conclusions

We are the first to examine the associations between anti-IFN-γ autoAbs and HZ development in RA patients treated with JAKis. The results revealed significantly lower MCP-1 levels in RA patients with HZ than those without, and significantly higher titers of anti-IFN-γ IgG in patients with severe HZ compared to those with non-severe HZ. Nevertheless, these associations were observed in a small cohort of RA patients and require further validation.

## Figures and Tables

**Table 1:** Demographic data, clinical characteristics, laboratory findings, and the used medications in RA patients with and without new-onset herpes zoster (HZ) ^#^.

Characteristics	RA with HZ (*n* = 24)	RA without HZ (*n* = 42)	*p*-Value
Age at study entry, years	58.7 ± 7.3	59.2 ± 7.7	0.995
Female, *n* (%)	18 (75.0%)	34 (81.0%)	0.755
RA disease duration, years	11.2 [9.3–13.8]	9.8 [7.8–10.7] *	0.020
Prior HZ history, *n* (%)	7 (29.2%)	5 (11.9%)	0.103
Baseline ESR, mm/h	45.0 [31.0–77.6]	44.0 [33.0–69.8]	0.743
Baseline C-reactive protein, mg/dL	1.37 [0.39–4.12]	2.17 [1.2–3.5]	0.126
Baseline DAS28 score	6.16 [5.51–6.71]	6.63 [6.01–7.16]	0.053
RF-positivity, *n* (%)	18 (75.0%)	33 (78.6%)	0.767
ACPA-positivity, *n* (%)	19 (79.2%)	29 (69.0%)	0.767
Concomitant steroids dose, mg/day	5.0 [5.0–6.0]	4.0 [2.0–4.0] ***	<0.001
Use of steroids ≧ 5 mg/day, *n* (%)	21 (87.5%)	4 (9.5%) ***	<0.001
JAKi monotherapy, *n* (%)	10 (41.7%)	15 (37.5%)	0.792
Concomitant csDMARDs, *n* (%)			
Methotrexate	8 (33.3%)	8 (19.0%)	0.238
Hydroxychloroquine	6 (25.0%)	19 (45.2%)	0.121
Leflunomide	2 (8.3%)	4 (9.5%)	1.000
The used JAKi			
Tofacitinib, *n* (%)	19 (38.0%)	31 (62.0%)	NA
Baricitinib, *n* (%)	1 (25.0%)	3 (75.0%)	NA
Upadacitinib, *n* (%)	4 (33.3%)	8 (66.7%)	NA
Current smoker, *n* (%)	5 (20.8%)	4 (9.5%)	0.268

^#^ Data were expressed as mean ± SD or number (%). NA: not applicable; RA, rheumatoid arthritis; ESR, erythrocyte sedimentation rate; DAS28, the 28-joint disease activity score; RF, rheumatoid factor; ACPA, anti-citrullinated peptide antibodies; JAKi, Janus kinase inhibitor; csDMARDs, conventional synthetic disease-modifying anti-rheumatic drugs. * *p* < 0.05, *** *p* < 0.001, HZ (+) vs. HZ (−) as determined using the chi-squared test for between-group differences in categorical variables or by Fisher exact *t*-test for between-group comparison of numerical variables. Statistically significant differences are marked in bold.

## Data Availability

The raw data supporting the conclusions of this article will be made available by the authors on request.
